# Dynamic Deformation Reconstruction of Variable Section WING with Fiber Bragg Grating Sensors

**DOI:** 10.3390/s19153350

**Published:** 2019-07-30

**Authors:** Zhen Fu, Yong Zhao, Hong Bao, Feifei Zhao

**Affiliations:** 1 Key Laboratory of Electronic Equipment Structure Design of Ministry of Education, Xidian University, Xi’an 710071, China; 2 School of New Energy Vehicles, Henan Mechanical and Electrical Vocational College, Zhengzhou 451191, China

**Keywords:** deformation reconstruction, fuzzy system, inverse finite element method, strain modification, variable section wing

## Abstract

In order to monitor the variable-section wing deformation in real-time, this paper proposes a dynamic reconstruction algorithm based on the inverse finite element method and fuzzy network to sense the deformation of the variable-section beam structure. Firstly, based on Timoshenko beam theory and inverse finite element framework, a deformation reconstruction model of variable-section beam element was established. Then, considering the installation error of the fiber Bragg grating (FBG) sensor and the dynamic un-modeled error caused by the difference between the static model and dynamic model, the real-time measured strain was corrected using a solidified fuzzy network. The parameters of the fuzzy network were learned using support vector machines to enhance the generalization ability of the fuzzy network. The loading deformation experiment shows that the deformation of the variable section wing can be reconstructed with the proposed algorithm in high precision.

## 1. Introduction

Through the real-time perception of the deformation of the wing structure, the flight attitude is adjusted in real time by the actuation and control systems to ensure the safety of the aircraft. This is one of the key technologies for the next generation of intelligent space vehicles. Using the strain sensors arranged on the wing structure to obtain the strain data of the structure in real time, and reconstructing the deformation information of the structure using the specific strain displacement relation [1], that is, shape sensing, it has become a research hotspot of the wing structure deformation. Due to the advantages of small size, anti-electromagnetic interference and the ability to form a large-scale quasi-distributed sensing network [2], the FBG strain sensors are widely used in the field of shape sensing.

The key to shape sensing is constructing a relationship between the structural deformation and the strain measurement. There are many research methods of strain-based deformation reconstruction proposed by domestic and foreign scholars, such as the modal transformation method, piecewise linearization method, and finite element method. The modal transformation method can accurately reconstruct the deformation of plate and beam structure [3], but it needs accurate finite element model. Another problem is that the algorithm is difficult to apply to the large deformation of geometric nonlinear structures because it is based on the principle of linear superposition [4]. The Ko method is based on the classical Euler Bernoulli beam theory [5]. By integrating the discrete surface strain measurements with piecewise continuous polynomials, high-precision reconstruction of the beam element in a one-dimensional direction can be achieved. The Ko method can be used for deformation reconstruction of cantilever beam structure, and ground experiment for deformation of wing structure has been realized [5,6]. The scheme is only suitable for one-dimensional deformation of the beam structure, but it is difficult to estimate the element deformation under multidimensional complex loads because the scheme requires a large number of strain sensors.

In order to meet the requirements of deformation monitoring, the deformation reconstruction algorithm should be able to adapt to complex topology and boundary conditions, and maintain accuracy and stability under a wide range of loads or elastic inertia changes of materials. Tessler and Spangler have proposed an inverse finite element method (iFEM) [7], which is based on the least square variational principle and uses the measured strain data to estimate the deformation of plate structure [8,9,10]. Furthermore, they have applied it to shape sensing of plate and shell structure undergoing large displacements [11]. Based on inverse finite element theory, Gherlone realized static deformation reconstruction of wing plate structure [12]. Furthermore, the inverse finite beam element, which is suitable for beam/frame structure deformation, is constructed by combining iFEM with Timoshenko beam theory, and the three-dimensional deformation reconstruction of frame structure under static condition is realized [4]. Based on the theory of ZigZag and the idea of inverse finite element, Cerracchio constructed the deformation reconstruction model of plate element for composite sandwich structure [13]. Yong Zhao et al. have studied the effect of sensor distribution on the reconstruction accuracy in the process of inverse finite beam element reconstruction, and realized the 3D deformational reconstruction of the wing like frame structure [14,15,16]. Xinglin Pan et al. have deeply studied the effect of measurement strain error on reconstruction accuracy in the process of static deformation reconstruction of beam structure, and put forward the error correction using fuzzy network [17]. When the wing is regarded as a constant-section beam structure, the above research studies the deformation reconstruction of the wing structure with high precision [18,19]. For some variable-section wings, when the element division is dense enough, the partial element can be regarded as a constant-section beam, but the number of sensors used is increased, and furthermore, the error caused by element superposition is so big that it cannot be neglected [20]. Guanghong Chuan et.al analyzed the stiffness matrix error of the variable-section Timoshenko Beam for the different rates of the section areas [21]. With the above conclusion, the reconstruction model of the cross-section wing is constructed in this paper. Meanwhile, the above studies only focus on the structural deformation reconstruction under static load, but not on the structural deformation reconstruction under dynamic excitation. Furthermore, it is found that the reconstruction accuracy and stability of inverse finite element method will be affected by the accuracy of the deformation reconstruction model and the sensor placement.

For the sake of sensing the deformation of the cross-section wing accurately, a variable cross-section inverse finite beam element model is proposed in this paper. Meanwhile, this paper derives the relationship between strain and deformation of the cross-section wing based on inverse finite element method. The deformation reconstruction of a cross-section wing is accomplished with the strain measurement of the structure surface. Furthermore, in order to remove the influence of the strain sensors installation and dynamic un-modeled error for the dynamic deformation reconstruction, this paper proposes a self-structuring linear support vector regression algorithm fuzzy network (SSILSVRFN) to modify the strain measurements. The inverse finite element method is employed to reconstruct the deformation displacement with the modified strain, and the accuracy of the deformation reconstruction is improved. The content of this paper is divided into the following parts: firstly, based on the inverse finite element framework, the inverse finite element reconstruction equation for the variable cross-section beam is constructed for the deformation reconstruction of glass fiber wing model with variable cross section. Then, a fuzzy network based on iterative linear support vector regression is constructed to correct the real-time error of dynamic strain measurement. Finally, the vibration experiments on the fiberglass wing model show that the proposed algorithm can effectively improve the accuracy and stability of wing deformation reconstruction.

## 2. A Deformation–Reconstruction Model for Wing with Variable Cross-Section

### 2.1. Inverse Finite Element Model for Variable Cross-Section Beam

**Figure 1:**
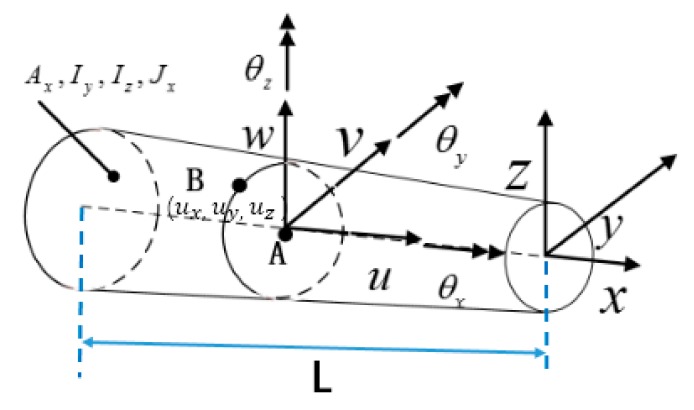
Schematic diagram of a variable section beam.

For a typical variable-section beam (Figure 1), the displacement of point B on the beam surface can be expressed with six kinematic variables $\left[u,v,w,\theta_{x},\theta_{y},\theta_{z}\right]$ on point A [4]

(1) $$u_{x}\left(x,y,z\right)=u\left(x\right)+z\theta_{y}\left(x\right)-y\theta_{z}\left(x\right)\;u_{y}\left(x,y,z\right)=v\left(x\right)-z\theta_{x}\left(x\right)\;u_{z}\left(x,y,z\right)=w\left(x\right)+y\theta_{x}\left(x\right)$$

where $u_{x},u_{y},$ and $u_{z}$ are the displacements along the x,y, and *z* axes, respectively, with $u\left(x\right),v\left(x\right),$ and $w\left(x\right)$ denoting the displacements at *y = z =* 0; $\theta_{x}\left(x\right)$,$\;\theta_{y}\left(x\right),$ and $\;\theta_{z}\left(x\right)$ are the rotations about the three coordinate axes; positive orientations for the displacements and rotations are depicted in Figure 1. These kinematic assumptions neglect the effect of axial warping due to torsion, i.e., each cross-section remains flat and rigid with respect to thickness-stretch deformations along the *y* and *z*-axes. The six kinematic variables can be grouped in vector form as

(2) $$u\left(x\right)={\left[u,v,w,\theta_{x},\theta_{y},\theta_{z}\right]}^{T}$$

In the finite element framework, the deformation of any point along the centroid-axis of the beam element can be obtained by interpolation of the determined shape function $N\left(x\right)$ and the node degrees of freedom $\;u^{\varepsilon }$.

(3) $$u\left(x\right)=N\left(x\right)u^{\varepsilon }$$

Based on the small strain hypothesis, the relationship between the section strain at any point along the centroid-axis and the node degree of freedom is

(4) $$e\left(u\right)=B\left(x\right)u^{\varepsilon }$$

where the matrix $B\left(x\right)$ (see Appendix A) contains the derivatives of the shape functions $N\left(x\right)$, and the section strain vector $e\left(u\right)$ is expressed as

(5) $$e\left(u\right)\equiv {\left[e_{1},e_{2},e_{3},e_{4},e_{5},e_{6}\right]}^{T}$$

$$e_{1}\left(x\right)\equiv u_{\;,x}\left(x\right)\;\;\;e_{4}\left(x\right)\equiv w_{\;,x}\left(x\right)+\theta_{y}\left(x\right)$$

$$e_{2}\left(x\right)\equiv \theta_{y,x}\left(x\right)\;\;\;e_{5}\left(x\right)\equiv v_{\;,x}\left(x\right)-\theta_{z}\left(x\right)$$

$$e_{3}\left(x\right)\equiv -\theta_{z,x}\left(x\right)\;\;\;e_{6}\left(x\right)\equiv \theta_{x,x}\left(x\right)$$

And the surface strain vector [ $\varepsilon_{x}\left(x,y,z\right),\gamma_{xz}\left(x,y\right),\gamma_{xy}\left(x,y\right)$ ] *^T^* of the beam element can be expressed with the above section strain vector $e\left(u\right)$ in theory:

(6) $$\varepsilon_{x}\left(x,y,z\right)=e_{1}\left(x\right)+ze_{2}\left(x\right)+ye_{3}\left(x\right)\;\gamma_{xz}\left(x,y\right)=e_{4}\left(x\right)+ye_{6}\left(x\right)\;\gamma_{xy}\left(x,y\right)=e_{5}\left(x\right)-ze_{6}\left(x\right)$$

While, the section strain vector $e\left(u\right)$ is calculated from the kinematic variables and cannot be directly measured. In the inverse finite element method, the least square error between the section strain $e\left(u\right)$ and the section strain $e^{\varepsilon }$ obtained from the surface measured strain is constructed as following

(7) $$\emptyset \left(u\right)=||e\left(u\right)-e^{\varepsilon }{||}^{2}$$

when $\emptyset \left(u\right)$ obtains the minimum value, the section strain $e\left(u\right)$ is replaced with the section strain $\;e^{\varepsilon }$ to form a relationship model between the beam element node degree of freedom and the section strain

(8) $$k^{e}u^{e}=f^{\varepsilon }$$

where $k^{e}=\frac{L}{n}*\sum_{i=1}^{n}\left[B^{T}\left(x_{i}\right)B\left(x_{i}\right)\right]$, $f^{\varepsilon }=\frac{L}{n}*\sum_{i=1}^{n}\left[B^{T}\left(x_{i}\right)e^{\varepsilon i}\right]$. Note that $k^{e}$ resembles an element stiffness matrix of the direct finite element method and $f^{\varepsilon }$ resembles the load vector; *L* is the element length (Figure 1); n and $x_{i}$ ($0\leq x_{i}\leq L$) are, respectively, the number and the axial coordinate of the locations where the section strains are evaluated, and the superscript εi is used to denote the section strains computed from the surface measured strain.

### 2.2. Calculation of Section Strain of Variable Section Beam Element

**Figure 2:**
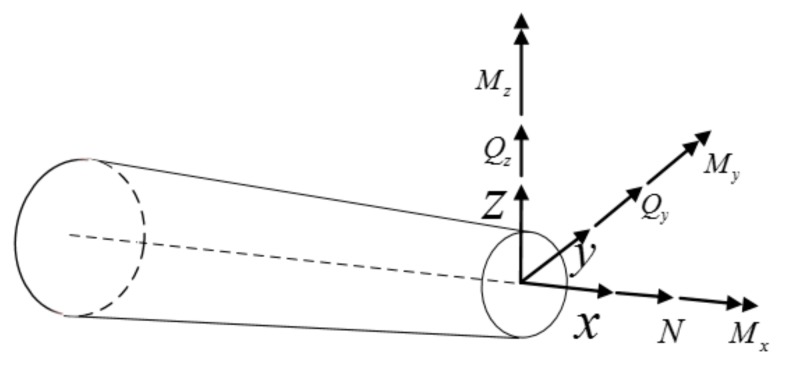
The force form of the beam.

Correctly solving the section strain is the key to the element deformation reconstruction. For the constitutive cross-section beam (Figure 2), the relationship between the section strain $e^{\varepsilon }$ and the external load force and moment can be expressed as [4]

(9) $$\begin{array}{l} N=A_{x}e_{1}{}^{\varepsilon }\;M_{x}=J_{x}e_{6}{}^{\varepsilon }\\ Q_{y}=G_{y}e_{5}{}^{\varepsilon }\;M_{y}=D_{y}e_{2}{}^{\varepsilon }\\ Q_{z}=G_{z}e_{4}{}^{\varepsilon }\;M_{z}=D_{z}e_{3}{}^{\varepsilon } \end{array}$$

where $A_{x}\equiv EA\;$ is the axial rigidity;$\;G_{y}\equiv k_{y}^{2}GA$ and $G_{z}\equiv k_{z}^{2}GA$ are the shear rigidities, with $k_{y}^{2}\;$ and $\;k_{z}^{2}$ denoting the shear correction factors; $J_{x}\equiv GI_{p\;}$ is the torsional rigidity; $D_{y}\equiv EI_{y\;}$ and $D_{z}\equiv EI_{z\;}$ are the bending rigidities; A  is the section area; E (Young’s modulus) and G (shear modulus) are the elastic constants. *M* is a moment acting in a certain direction on the unit, and its unit is N*m; the unit of *Q* is *N*.

Once the type of the external load is known, the format of the section strain $e^{\varepsilon }$ can be determined with using Equation (9). For example, the concentrated load, bending moment and torsion performed on the beam element, the in-plane force $N,Q_{y},Q_{z}\;$ and torsion $M_{x}\;$ of the element are constant along the axis direction, and the bending moment $\;M_{y}\;$ and $M_{z\;}$ change along the *x*-axis. The conclusion is valid for both constant-section beam and variable-section beam [21]. The section strain $e_{1}^{\varepsilon }$ can be expressed as

(10) $$e_{1}^{\varepsilon }\left(x\right)=\frac{C_{1}}{A_{x}\left(x\right)}$$

where $C_{1}$ is an unknown constant and $A_{x}\left(x\right)$ is the tensile stiffness of the element section. Similarly, the section strains $e_{4}^{\varepsilon }$,$\;e_{5}^{\varepsilon },$ and $e_{6}^{\varepsilon }$ can be written as follows

(11) $$\begin{array}{l} e_{4}{}^{\varepsilon }(x)=\frac{C_{4}}{G_{z}(x)}\\ e_{5}{}^{\varepsilon }(x)=\frac{C_{5}}{G_{y}(x)}\\ e_{6}{}^{\varepsilon }(x)=\frac{C_{6}}{I_{p}(x)} \end{array}$$

where $G_{y}\left(x\right),G_{z}\left(x\right),\mathrm{and}\;I_{p}\left(x\right)$ are the bending stiffness and torsional stiffness of the variable section beam element [21]; $C_{4}$, $C_{5}$, and $C_{6}$ are unknown constants; $C_{4}$ and $C_{5}$ can be computed with the following equation in [4]:

(12) $$\begin{array}{l} C_{4}=Q_{y}=\frac{\partial D_{z}e_{3}^{\varepsilon }}{\partial x}\\ C_{5}=Q_{z}=\frac{\partial D_{y}e_{2}{}^{\varepsilon }}{\partial x} \end{array}$$

Since the changes of bending moment $\;M_{y\;}$ and $\;M_{z\;}$ along the centroid-axis of the beam element are linear, $M_{y\;}$ and $\;M_{z\;}$ can be assumed as

(13) $$\begin{array}{l} M_{y}(x)=C_{5}x+C_{2}\\ M_{z}(x)=C_{4}x+C_{3} \end{array}$$

where $C_{2}$ and $C_{3}$ are unknown constants. Submitting the Equation (13) into Equation (9), $e_{2}^{\varepsilon }$ and $e_{3}^{\varepsilon }$ can be expressed as

(14) $$e_{2}{}^{\varepsilon }(x)=\frac{C_{5}x+C_{2}}{D_{y}(x)}\;,\;e_{3}{}^{\varepsilon }(x)=\frac{C_{4}x+C_{3}}{D_{z}(x)}$$

**Figure 3:**
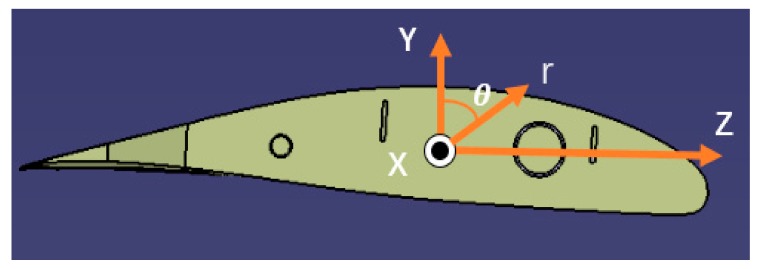
Orthogonal and cylindrical coordinate systems of the wing section.

For the variable-section wing (Figure 3), the relationship between the surface strain measurement $\varepsilon^{*}$ and the section strain vector is ascertained with using the strain-tensor transformation from the $\left(\theta ,X,r\right)$ to (*X*, *Y*, *Z*) [4,22,23]:

(15) $$\varepsilon^{*}=\varepsilon_{x}\left(cos^{2}\beta -\nu sin^{2}\beta \right)+\gamma_{x\theta }cos\beta sin\beta$$

Substituting $r=R_{i}$ in Equation (6), yields

$$\varepsilon_{x}=e_{1}+e_{2}R_{i}sin\theta +e_{3}R_{i}cos\theta$$

(16) $$\gamma_{x\theta }=e_{4}cos\theta -e_{5}sin\theta +e_{6}R_{i}$$

**Figure 4:**
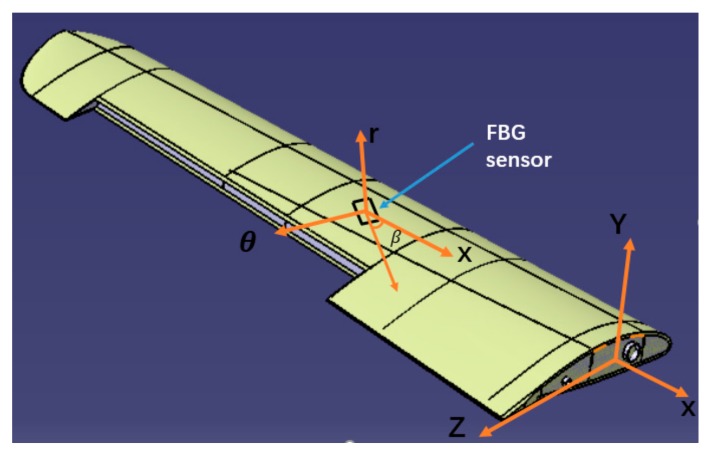
Location and coordinate system of a FBG sensor placed on the wing external surface.

Substituting (10), (11), (14), and (16) into (15) gives the relationship between the strain measurements and the section strain for the variable section beam element as (Figure 4)

(17) $$\begin{array}{ll} \varepsilon^{*}(x_{i},\theta_{i},\beta_{i}) & =\frac{C_{1}}{A_{x}(x_{i})}(\cos^{2}\beta_{i}-v\sin^{2}\beta_{i})+\frac{C_{5}x_{i}+C_{2}}{D_{y}(x_{i})}(\cos^{2}\beta_{i}-v\sin^{2}\beta_{i})R_{i}\sin\theta_{i}\\ & +\frac{C_{4}x_{i}+C_{3}}{D_{z}(x_{i})}(\cos^{2}\beta_{i}-v\sin^{2}\beta_{i})R_{i}\cos\theta_{i}+\frac{C_{4}}{G_{z}(x_{i})}\cos\beta_{i}\sin\beta_{i}\cos\theta_{i}\\ & -\frac{C_{5}}{G_{y}(x_{i})}\cos\beta_{i}\sin\beta_{i}\sin\theta_{i}+\frac{C_{6}}{I_{p}(x_{i})}R_{i}\cos\beta_{i}\sin\beta_{i} \end{array}$$

where $R_{i}$=$\sqrt[{2}]{y_{i}^{2}+z_{i}^{2}}$; ν is Poisson’s ratio; $R_{i}$ is the polar radius of the beam section; $x_{i},\theta \;$ and β are the positions of the strain measurements on the surface of the beam element.

Therefore, the unknown parameters $C_{1},C_{2},C_{3},C_{4},C_{5},C_{6}$ can be solved from six different surface measurement strain values $x_{i},\theta_{i},\beta_{i},\left(i=1,\ldots ,6\right)$ with using the Equation (17), and the section strains $e^{\varepsilon \;}$ will be determined with Equations (10), (11), and (14).

## 3. Strain Error Correction

Because of the strain measurement system error and the model error, there is a big deviation between the actual displacement and the displacement computed from the strain measurement data with iFEM. The strain measurement system error consist of the location error resulted from the FBG sensors attachment process, measurement error of strain measurement instrument et al. The model error, including the dynamic un-modeled error caused by the difference between the static model and dynamic model, the connection and segmentation of the structure elements. For removing the above errors, the basic modification strategy is: (1) the actual strain value $\hat{\varepsilon }$ is computed from actual deformation captured from the third-party measurement instrument with reconstruction model, Equation (8). (2) the fuzzy network of the self-structuring iterative linear support vector regression fuzzy network (SSILSVRFN) algorithm [24] is trained and fixed with the above actual strain value $\hat{\varepsilon }$ and the corresponding measured strain values ε. (3) The actual deformation of the structure for any loading cases can be computed from the actual strain value $\hat{\varepsilon_{A}}$ computed from the corresponding strain measurement $\varepsilon_{A}$ with the above fixed fuzzy network. 

For the deformation reconstruction based on strain measurement, there are many error factors in the measurement of structural surface strain. Thus, the difference exists between the measured strain and the actual strain; and the corresponding relationship between the measured strain and the actual strain is difficult to be accurately described with mathematical expressions. The self-structuring fuzzy network (SSFN) is attempted to approach this unknown relationship because the fuzzy network has the characteristic of infinite approximation to any function mapping relation. SSFN algorithm constructs the network from zero rule, and the adjustment of the rules number and structure is based on the training data as current network input. The training network is difficult to achieve the desired application effect in the testing phase because network structure is optimal for the current data. This paper proposes a SSILSVRFN using the support vector regression theory and cluster idea. The SSILSVRFN system is divided into the structure training phase and the parameter learning phase.

The structure training phase adopts the SSRG (self-structuring rule generation) algorithm for automatic fuzzy rule generation and initialization. According to the spatial distribution of the input data and the analysis of the overall data, a reasonable distribution of fuzzy sets center and width is achieved. Not only the size of the whole network is effectively reduced, but also the impact on the network structure of the data order is avoided.

**Figure 5:**
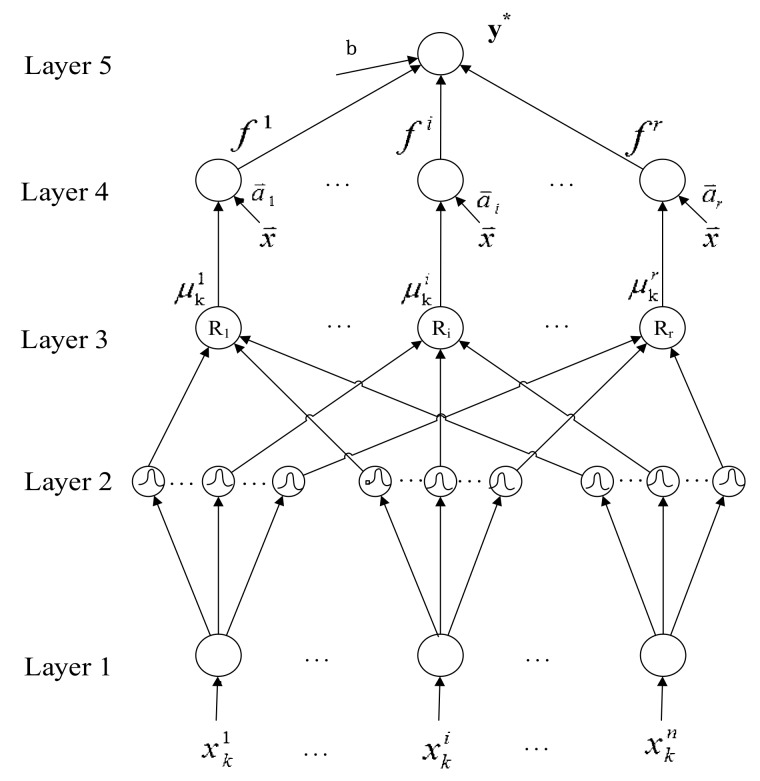
Structure of the self-structuring linear support vector regression algorithm fuzzy network (SSILSVRFN).

The SSILSVRFN proposed in this paper is divided into five layers, as shown in Figure 5.

In parameter learning phase, the support vector regression (SVR) is based on the structural risk minimization principle; and SVR learning algorithm has a better performance in generalization and prevention of over-fitting. Therefore, SSILSVRFN adopts iterative linear SVR (ILSVR) to iteratively adjust parameter of fuzzy rules.

**Figure 6:**
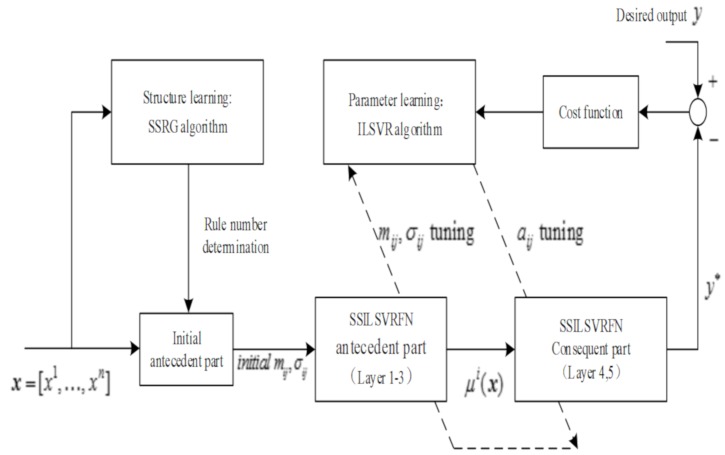
The SSILSVRFN training.

The SSILSVRFN training and learning system diagram is shown in Figure 6. 

### 3.1. SSILSVRFN Structure Learning

The structure learning determines the fuzzy rule number and initial fuzzy set center $m_{ij}$ and width $\sigma_{ij}$. Once the number of fuzzy rules is determined, the numbers of nodes in the layer 2, 3 and 4 will be accordingly determined, which are *nr*, *r* and *r*, respectively. In the structure learning phase, a fuzzy rule is regarded as a cluster that corresponds to a rule node of layer 3 in the input space. A SSRG algorithm is proposed to determine the suitable number of rules.

**Figure 7:**
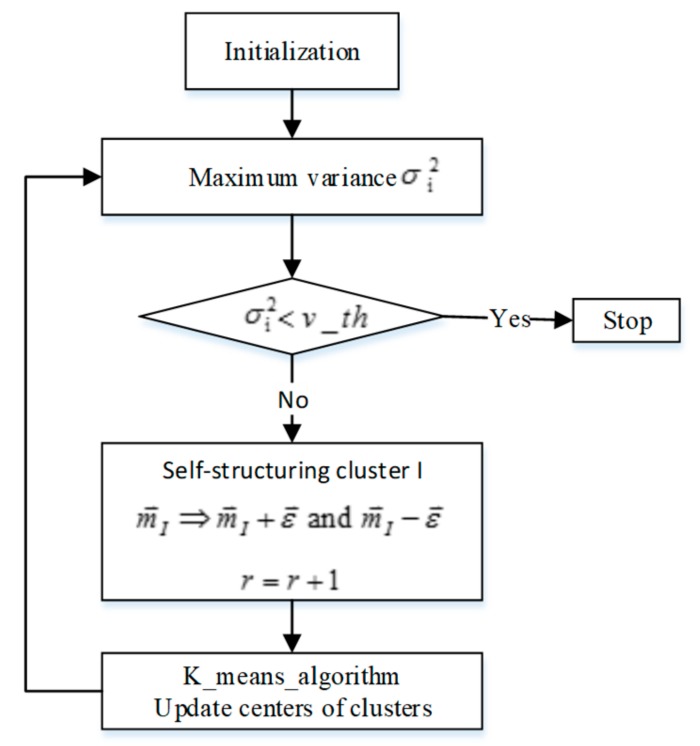
The flow chart of self-structuring rule generation SSRG.

The flow chart (Figure 7) is as follows

The algorithm steps are presented as follows 

Step 1: classify the training samples, and set the number of iteration $\;N_{ite}=0$. Assign each input training data *x* to cluster P, which is calculated as follows

(18) $$P=\arg\underset{1\leq p\leq r}{\min}d\left(x,m_{p}\right)$$

(19) $$d\left(x,m_{p}\right)=\sum_{j=1}^{n}\left|x^{j}-m_{pj}\right|$$

Step 2: calculate the maximum variance. For the first cluster, the variance is defined as follows

(20a) $$I=\arg\underset{1\leq i\leq r}{\max}{\bar{\sigma }}_{i}^{2}$$

(20b) $${\bar{\sigma }}_{i}^{2}=\sum_{j=1}^{n}{\bar{\sigma }}_{ij}^{2}$$

(20c) $${\bar{\sigma }}_{ij}^{2}=\frac{1}{N_{i}}\sum_{x\in C_{i}}{\left(x^{j}-m_{ij}\right)}^{2}$$

Step 3: if the maximum value of cluster variance ${\bar{\sigma }}_{I}^{2}$ is less than the threshold value $v_{th}$ and the current iteration number is less than 20, stop; otherwise, the cluster with the largest difference will be split to generate a new cluster. The center point vectors of the new clusters are, respectively, set as $m_{I}+\varepsilon$ and $m_{I}-\varepsilon$, where ε is a constant vector with a small value. This paper sets the ith component of ε to be 1% of the domain of input variable $x^{i}$.

Step 4: update the centers of clusters. Recalculate the center of cluster **P** as

(21) $$m_{P}\leftarrow m_{P}+\frac{1}{N_{P}}\sum_{k=1}^{N_{P}}\left(x_{k}-m_{P}\right)$$

where $N_{p}$ is the number of samples in cluster **P**. The iteration number is updated as $\;N_{ite}=N_{ite}+1$.

### 3.2. SSILSVRFN Parameter Learning

The iterative linear SVR (ILSVR) learning algorithm is used to adjust the antecedent and consequent parameters of fuzzy rules in the SSILSVRFN algorithm. In ILSVR algorithm, the purpose of learning parameters is to optimize antecedent and consequent parameters of fuzzy rules based on the cost function.

#### 3.2.1. Consequent Parameter Learning

In this section, the fourth layer represented by the structure diagram in Figure 5 is used to calculate the output value of each rule. The calculation expression is as follows

(22) $$f^{i}=\mu^{i}(x)\cdot \left(a_{i0}+\sum_{j=1}^{n}a_{ij}x^{j}\right)=\mu^{i}(x)\cdot \sum_{j=0}^{n}a_{ij}x^{j},x^{0}\underline{\underline{\Delta }}\;1$$

where $a_{i0}+\sum_{j=0}^{n}a_{ij}x^{j}$ is the corresponding consequent value of the node; and $\mu^{i}\left(x\right)$ is the corresponding firing strength.

The fifth layer represents the output variable of the output layer, which calculates as follows

(23) $$y^{*}=\sum_{i=1}^{r}f^{i}+b$$

where *b* is the compensation constant and $f^{i}$ is the output value of the node.

By substituting (22) into (23), the output of SSILSVRFN can be transformed as follows

(24) $$y^{*}(x)=\sum_{i=1}^{r}\sum_{j=0}^{n}a_{ij}\mu^{i}x^{j}+b$$

Equation (24) shows that the output $y^{*}$ is a linear function of $\mu^{i}x^{j}$ with weights $a_{ij}$. Therefore, linear SVR can be employed to learn parameters $a_{ij}$. After structure learning, the number of rules *r* and the initial rule antecedent part parameters are determined. The input data $x_{k}$ is transformed to the following vector

(25) $$\begin{array}{ll} \varphi (x_{k}) & =[\varphi^{1}(x_{k})\;,\;\ldots \;,\;\varphi^{r(n+1)}(x_{k})]\\ & =\left[\mu^{1}x_{k}^{0}\;,\;\ldots \;,\;\mu^{1}x_{k}^{n}\;,\;\ldots \;,\;\mu^{r}x_{k}^{0}\;,\;\ldots \;,\;\mu^{r}x_{k}^{n}\right]\in {ℜ}^{r(n+1)} \end{array}$$

The vector φ as input into a linear SVR, and the training data pairs are represented as follows

(26) $$\begin{array}{ll} S & =\left\{\begin{array}{cccc} (x_{1},y_{1}), & (x_{2},y_{2}), & \cdots , & \left(x_{N},y_{N}\right) \end{array}\right\}\\ & =\left\{\begin{array}{cccc} \left(\varphi (x_{1}),y_{1}\right), & \left(\varphi (x_{2}),y_{2}\right), & \ldots , & \left(\varphi (x_{N}),y_{N}\right) \end{array}\right\} \end{array}$$

The linear regression function $y^{*}(x)$ is given as

(27) $$y^{*}(x)=\sum_{k=1}^{N}(\alpha_{k}-{\hat{\alpha }}_{k})x_{k}x+b$$

According to Equations (26) and (27), the optimal linear regression function $y^{*}(x)$ is given as

(28) $$y^{*}(x)=\sum_{k=1}^{N}(\alpha_{k}-{\hat{\alpha }}_{k})\langle \begin{array}{cc} \varphi (x), & \varphi (x_{k}) \end{array}\rangle +b$$

where $\alpha_{k}$ c and ${\hat{\alpha }}_{k}$ are calculated by SVR software, i.e., Library for Support Vector Machines (LIBSVM) in this section. Based on Equation (25), Equation (28) can be represented as follows

(29) $$\begin{array}{ll} y^{*}(x) & =\sum_{k=1}^{N}(\alpha_{k}-{\hat{\alpha }}_{k})\sum_{m=1}^{r(n+1)}\varphi^{m}(x)\varphi^{m}(x_{k})+b\\ & =\sum_{m=1}^{r(n+1)}\left[\sum_{k=1}^{N}\left(\alpha_{k}-{\hat{\alpha }}_{k}\right)\varphi^{m}(x_{k})\right]\varphi^{m}(x)+b\\ & =\sum_{i=1}^{r}\sum_{j=0}^{n}\left[\sum_{k=1}^{N}\left(\alpha_{k}-{\hat{\alpha }}_{k}\right)\mu^{i}x_{k}^{j}\right]\mu^{i}x^{j}+b \end{array}$$

Since Equation (29) is equivalent to Equation (24), by comparing these two expressions, the mathematical expression of the parameters $a_{ij}$ can be obtained.

(30) $$a_{ij}=\sum_{k=1}^{N}\left(\alpha_{k}-{\hat{\alpha }}_{k}\right)\mu^{i}x_{k}^{j},i=1,\ldots ,r,j=0,\ldots ,n$$

#### 3.2.2. Antecedent Parameter Learning

The initial parameters $m_{ij}$ and $\sigma_{ij}$ in SSILSVRFN are determined by the SSRG algorithm. Further, these parameters are tuned based on the minimization of the cost function. The output function of SSILSVRFN in Equation (24) can be written as follows

(31) $$y^{*}(x)=\sum_{i=1}^{r}\left\{\exp\left[-\sum_{j=1}^{n}{\left(\frac{x^{j}-m_{ij}}{\sigma_{ij}}\right)}^{2}\right]\right\}\cdot \left(\sum_{j=0}^{n}a_{ij}x^{j}\right)+b$$

The above equation shows that the antecedent parameters $m_{ij}$ and $\sigma_{ij}$ are not the linear combination coefficients of the fuzzy network output $y^{*}$. Therefore, the linear SVR cannot be directly applied. In response to this problem, this section uses Taylor series expansion to linearize it. Since the parameters $m_{ij}$ and $\sigma_{ij}$ are independent of each other, the fuzzy network output $y^{*}$ is expanded as shown below.

(32) $$\begin{array}{ll} y^{*}(p) & =y^{*}\left(p(0)\right)+\sum_{i=1}^{r}\sum_{j=1}^{n}\Delta m_{ij}\left(m_{ij}-m_{ij}(0)\right)\\ & +\sum_{i=1}^{r}\sum_{j=1}^{n}\Delta \sigma_{ij}\left(\sigma_{ij}-\sigma_{ij}(0)\right)+y_{h}^{*} \end{array}$$

where $y_{h}^{*}$ is the remainder of the Taylor expansion, $P=\left[m_{11},\ldots ,m_{rn},\;\sigma_{11},\ldots \sigma_{rn}\right]$ denotes the antecedent parameters vector. The first partial derivative $∆m_{ij}$ and $∆\sigma_{ij}$ are, respectively, written as follows

(33) $$\Delta m_{ij}=\frac{\partial y^{*}}{\partial m_{ij}}=f^{i}\cdot 2\cdot \frac{x_{j}-m_{ij}}{{\left(\sigma_{ij}\right)}^{2}}$$

(34) $$\Delta \sigma_{ij}=\frac{\partial y^{*}}{\partial \sigma_{ij}}=f^{i}\cdot 2\cdot \frac{{\left(x_{j}-m_{ij}\right)}^{2}}{{\left(\sigma_{ij}\right)}^{3}}$$

Let ${\hat{y}}^{*}=y^{*}(p)-y^{*}(p(0))$. Equation (32) can be further transformed as follows

(35) $${\hat{y}}^{*}\approx \sum_{i=1}^{r}\sum_{j=1}^{n}\left[\Delta m_{ij}\left(m_{ij}-m_{ij}(0)\right)+\Delta \sigma_{ij}\left(\sigma_{ij}-\sigma_{ij}(0)\right)\right]$$

Equation (35) shows that ${\hat{y}}^{*}$ is a linear function with linear combination coefficients $m_{ij}-m_{ij}\left(0\right)$ and $\sigma_{ij}-\sigma_{ij}\left(0\right)$. Therefore, linear SVR can be used to optimize tuning parameters $m_{ij}-m_{ij}\left(0\right)$ and $\sigma_{ij}-\sigma_{ij}\left(0\right)$. Being similar to the derivation of consequent parameter learning, the parameters $m_{ij}$ and $\sigma_{ij}$ can be solved.

(36) $$\begin{array}{l} m_{ij}=m_{ij}(0)+\sum_{k=1}^{N}\left[\left(\alpha_{k}-{\hat{\alpha }}_{k}\right)\Delta m_{ij}(x_{k})\right]\\ \sigma_{ij}=\sigma_{ij}(0)+\sum_{k=1}^{N}\left[\left(\alpha_{k}-{\hat{\alpha }}_{k}\right)\Delta \sigma_{ij}(x_{k})\right],i=1,\ldots ,r,j=0,\ldots ,n \end{array}$$

The linearized function ${\hat{y}}^{*}$ in Equation (35) approximates the output of SSILSVRFN $y^{*}$ with the assumption that the updated values of $m_{ij}$ and $\sigma_{ij}$ are very close to the original $m_{ij}\left(0\right)$ and $\sigma_{ij}\left(0\right)$. After the optimization of the linear SVR, the updated parameters may be too large to meet this assumption. Thus, ILSVR is used to solve this problem. After several linear SVR learning iterations, the parameter learning tends to converge. The updated values of $m_{ij}$ and $\sigma_{ij}$ can meet the assumption in a Taylor expansion because the change in the two parameters tends to zero.

Throughout the learning process of parameters, the steps of the entire ILSVR algorithm can be summarized as follows

Step 1: set the maximal iteration number $T_{ite}$ and set the iteration index l to zero.

Step 2: calculate consequent parameters $\;a_{ij}\left(l\right)$, i=1,…,r,j=0,…,n by using the linear SVR and Equation (30).

Step 3: based on the learned parameters $a_{ij}\left(l\right)$ in Step 2, calculate the antecedent parameters $m_{ij}\left(l\right)$ and $\sigma_{ij}\left(l\right)$ using the linear SVR and Equation (36).

Step 4: if $\;l\geq T_{ite}$, this algorithm stops; otherwise, update the iteration index l=l+1 and go back to step 2.

**Figure 8:**
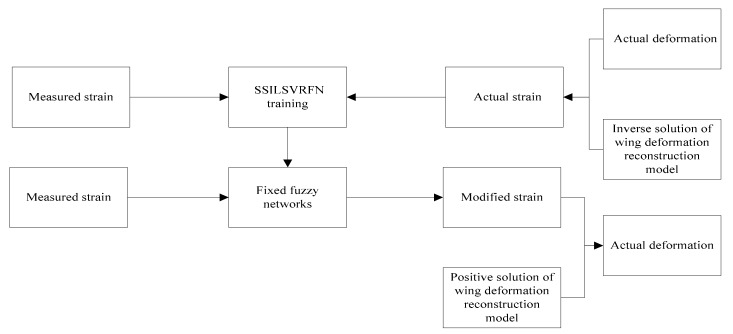
Structural block diagram of dynamic deformation reconstruction method for variable section wing.

The overall structure block diagram of the variable section wing dynamic deformation reconstruction method is shown in the Figure 8.

## 4. Verifications through Simulations and Experimentation

In order to assess the accuracy and effectiveness of the measurement scheme proposed in this paper, simulations and model experimentation are performed. In Section 4.1, a finite element model of a variable-section wing is constructed through direct finite element (FE) analyses software (ABAQUS), used to assess the accuracy of the reconstructing model of the variable-section wing. In Section 4.2, a physical wing model is tested under different dynamic loads, in order to assess the feasibility of measurement scheme proposed in this paper.

### 4.1. Simulation Test

**Figure 9:**
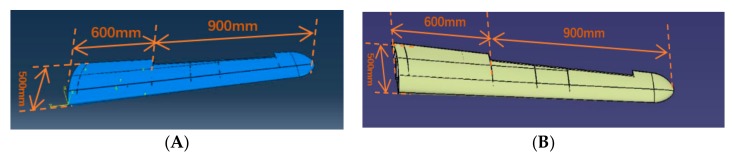
The finite element (FE) model and the CAD model of the variable section wing. (**A**) The FE model; (**B**) The CAD model.

**Figure 10:**
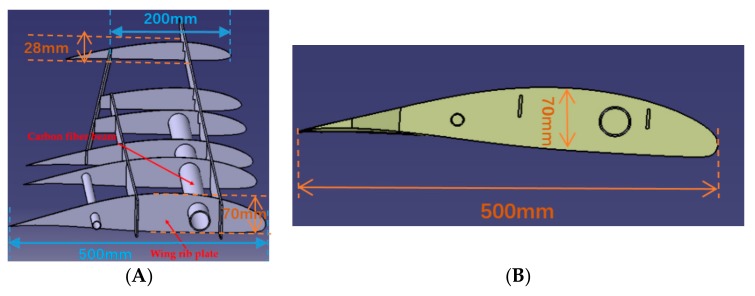
Wing structure. (**A**) Framework of the wing; (**B**) wing cross section.

The FE model of the whole variable section wing (Figure 9A) is directly obtained from the CAD model of the wing (Figure 9B) with using ABAQUS. The whole wing is combined the skin with the framework, and the framework mainly consists of glass fiber rib plate and glass fiber sheet. The single wingspan is 1500 mm long. The whole wing structure is divided into two segments for deformation reconstruction: the first section is the wing root part which is 600 mm; the second section is the aileron which is 900 mm. For the largest cross-sectional area of the wing root, the length and the width are 500 mm and 70 mm, respectively. For the smallest cross-sectional area of the wing tip, the length is 200 mm and the width is 28 mm (Figure 10). The cross section area decreases along the span direction, and the shape of each section is the similar (Figure 10B). For the wing skin, the Young’s modulus is E=8.3 GPa, the Poisson ratio is v=0.22, and the density is $\rho =4500\;\mathrm{kg}/m^{3}$. For the glass fiber rib plate and the glass fiber sheet, the Young’s modulus is E=7.3 GPa, the Poisson ratio is v=0.22, and the density is $\rho =5000\;\mathrm{kg}/m^{3}$. The wing is connected to the airframe with two thin-walled carbon fiber beams. For the carbon fiber beam, the Young’s modulus is E=210 GPa, the Poisson ratio is v=0.307, and the density is $\rho =4000\;\mathrm{kg}/m^{3}$. The longer beam length is 757 mm, the external radius is 18 mm  and the thickness is 2 mm, and the shorter beam length is 345 mm, the external radius is 7.5 mm  and the thickness is  1 mm. 

**Figure 11:**
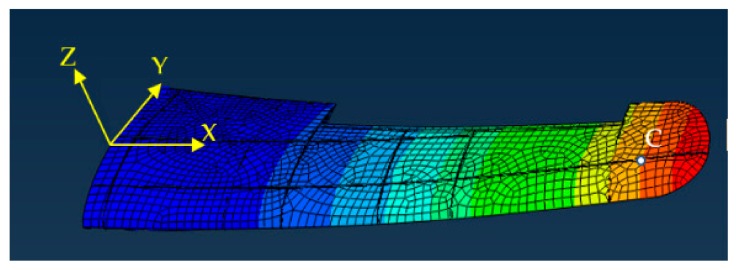
The contour plot of the wing deformation.

Two different loads are performed on the FE model. Herein, the point C (Figure 11) is selected as the target point to assess the accuracy of the reconstructing model. In Figure 11, the hexahedron element is used to divide the wing structure, and the total number of the element is 4546. The comparison between the deformation computed from the strain values with using the reconstruction model (Equation (7)) and the deformation analyzed with using the FE model for the point C is shown in Table 1. The strain values are obtained from the FE analysis. 

The comparisons of Table 1 demonstrate that the reconstructing model presents higher accuracy, for the main deformation $w_{z}$ under the two loading cases, the percent errors remain is below 6.0%. Although the percentage errors are relatively high for the displacements in the other two directions and the rotations for three directions, the absolute errors are very small. During the flight, the main deformation of the wing is the displacement along the vertical direction, Z (Figure 11).

### 4.2. Physical Model Test

**Figure 12:**
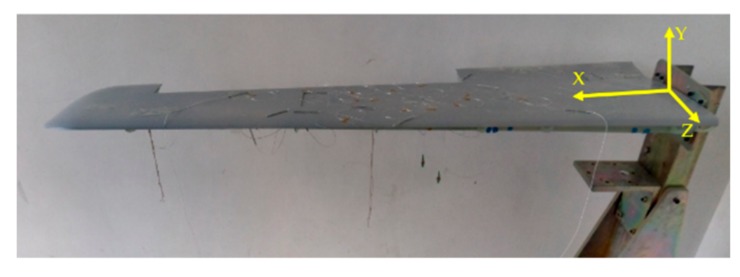
Variable-section wing physical model.

For the aim of assessing the effectiveness of the measurement scheme proposed in this paper, a dynamic loading test is performed on the variable-section wing physical model. The size of the physical wing (Figure 12) is same to the CAD model of the wing (Figure 9B).

**Figure 13:**
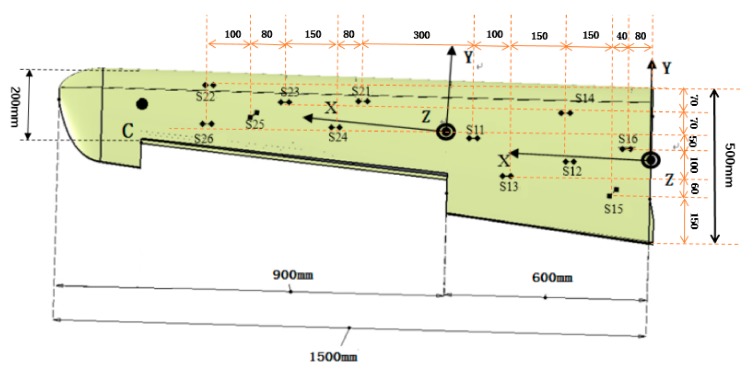
Finite element model of wing with variable cross section.

In the experiment, the strain data are obtained from the strain measurement system composed of FBG strain sensors (Fiber Bragg Grating| os1100, Micron Optics, Atlanta, GA, USA) and the FBG interrogator (Optical Sensing Instrument| Si 155, Micron Optics, Atlanta, GA, USA). Twelve FBG strain sensors (the range of initial wavelength is (1527 nm, 1564 nm)) are placed at different locations along the wing and used to capture the surface strain. The placement of the strain sensors is shown in Figure 13. 

**Figure 14:**
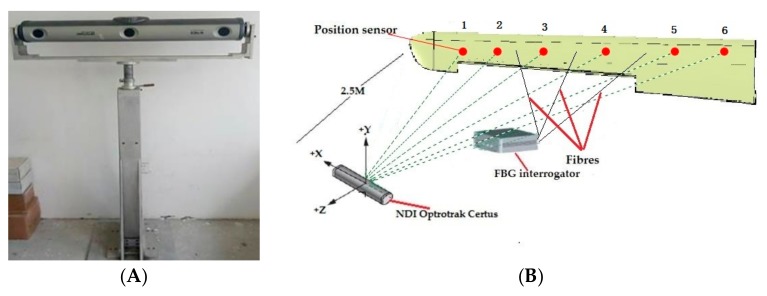
Wing tests. (**A**) NDI Optrotrak Certus; (**B**) experiment system.

In order to evaluate the accuracy of the deformation reconstructed from the algorithm proposed in this paper, the actual deformation of the wing structure is captured from the 3D optical measurement instrument (see Figure 14A, NDI Optrotrak Certus) which is abbreviated as NDI. Several position sensors which send the infrared lights to the CCD cameras of the NDI to reflect the deformation of the wing structure. The accuracy of NDI is 0.1 mm in its measurement range. The whole experiment system and the coordinate can be seen in Figure 14B.

**Figure 15:**
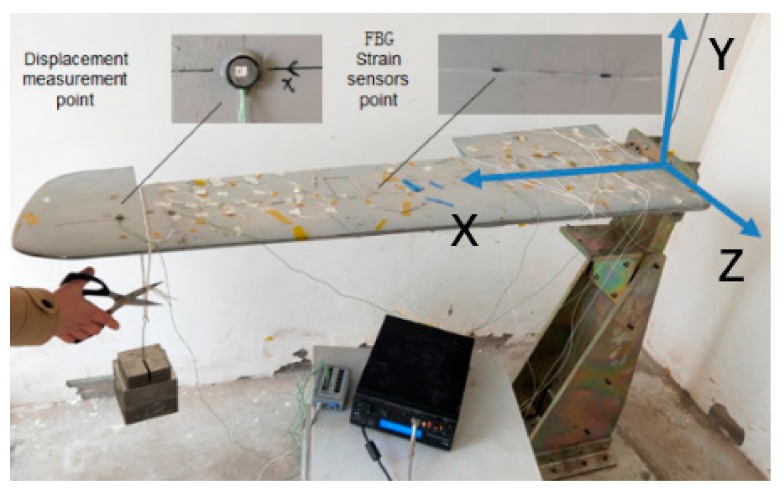
Dynamic load performed on the variable section wing model.

In the experiment, twenty different static loadings are performed on the end of the wing, which used for the fuzzy network training. The total weight of the loading is 20 kg. The dynamic load is caused by the sudden removal of the load applied on the wing tip (Figure 15). 

**Figure 16:**
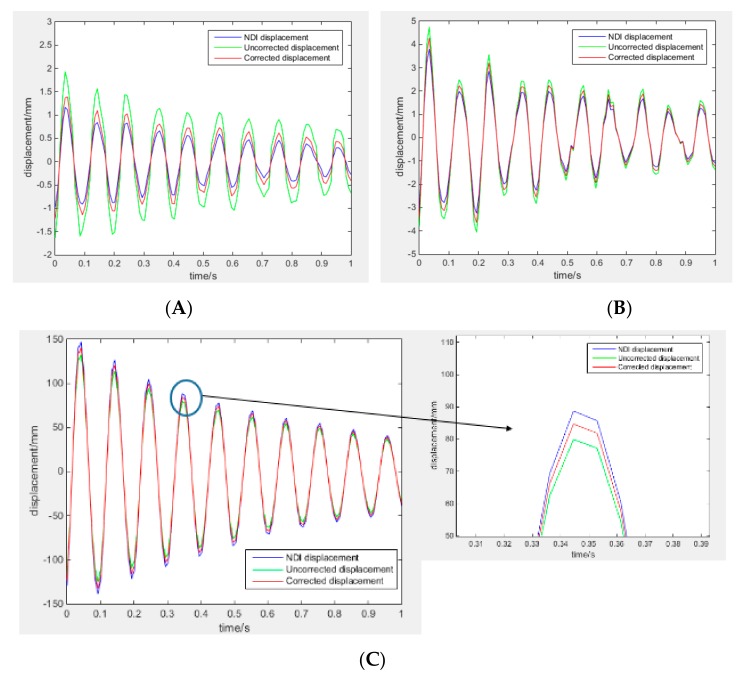
Comparison between deformation displacements of point C for the different time. (**A**) *X*-axis; (**B**) *Z*-axis; and (**C**) *Y*-axis.

Under the working condition, the aircraft is mainly subjected to the vertical lifting force. Therefore, in this experiment, the load along the *Y*-axis is mainly performed on the wing model. When the kinematic variable $u^{e}$ of the wing is solved by the measured strain through the Equation (8), any deformation of the wing surface can be calculated by using the Equation (1). To verify the accuracy of the reconstruction, the root mean square error RMS (Root Mean Square) is applied.

(37) $$\;RMS=\sqrt[{2}]{\sum_{i=1}^{j}{\left(disp^{NDI}\left(x_{i}\right)-disp^{iFEM}\left(x_{i}\right)\right)}^{2}/j}$$

where, $disp\left(x_{i}\right)\;$ is the displacement of one node along the wing in one direction; the superscript ‘NDI’ refers to the deformation values captured from NDI; ‘iFEM’ refers to the predicted values computed from strain data with iFEM; and *j* is the number of the nodes used to describe the wing deformation. In the test, the number of nodes used to describe the beam deformation is 6, and these nodes are placed on the surface of the wing (Figure 14B). Meanwhile, the node of maximum deformation, point C (Figure 13) is taken account. The tracking of point C for dynamic loading cases can be seen in Figure 16, and the comparison between the deformations of the point C computed from the strain data are shown in Table 2 and Table 3. The maximum percentage error among the individual nodal displacements is shown in Table 4. The comparison between the deformations of the whole wing computed from the strain data are shown in Table 5.

It is found that, the deformation compute from the modified strain data with iFEM on point C is close to the result captured from the NDI. From the Table 2, it is shown that the minimum value of the percentage error of the C point displacement is 3.9%, and the maximum value is 6.7% along the *y*-axis. Although the percentage errors are relatively high for the displacements in the other two directions, deformations along the *x*-axis and *z*-axis are very small from the Table 3. From the Table 4, it is shown that the maximum percentage error among the individual nodal displacements is 6.7%, and the maximum value is 4.1%.

From the Table 5, it is shown that the accuracy of the deformation reconstruction with iFEM is increased when the strain measurements are modified with using SSILSVRFN algorithm proposed in this paper. The RMS of the reconstruction for the maximum deformation is 12.01 mm when the strain measurements are unmodified, while RMS is 3.97 mm when the strain measurements are modified with using SSILSVRFN algorithm. The reduction percentage of RMS is 66.8% at least.

During the flight, the main deformation of the wing is the displacement along the vertical direction (Figure 11 and Figure 12). From the Table 1 and Table 2, numerical and experimental studies show that:

1. By comparing between the FE analysis and the reconstructing with using IFEM, numerical studies show that the percent error of the deformation reconstruction along the main direction remains below 6.0%.
2. Because of the strain measurement system error and the model error, experimental studies show that the percent error of the deformation reconstruction along the main direction computed from the unmodified strain measurements with iFEM remains below 13%.
3. Experimental application of the proposed method shows that: the percent error of the deformation reconstruction along the main direction computed from the modified strain measurements remains below 6.7%.

The dynamic deformation reconstruction algorithm proposed in this paper shows high precision for the wing structure with variable section.

## 5. Conclusions

In view of the effects of the dynamic un-modeled error and the sensor placement error on the cross-section wing deformation sensing, a dynamic deformation reconstruction algorithm for wing structure with variable section is proposed in this paper. For the sake of reducing the influence of the strain measurement error, the in situ strain data are modified with using SSILSVRFN in real time. The test performed on the wing shows that, the dynamic deformation of the variable cross-section wing can be accurately reconstructed from the strain measurement with the scheme proposed in this paper. The measurement scheme proposed in this paper can be applied to the dynamic deformation reconstruction of the structure from the variable section beam. If the measurement objects are different, the reconstruction model and the correction network need to be re-established. Nevertheless, the dynamic loading in this paper is caused by sudden removal of the load applied on the wing tip. The further work is performing a dynamic experiment on the shaking platform to simulate the dynamic deformation caused by different airflow excitations, to assess the effectiveness and accuracy of the reconstruction algorithm.

## Figures and Tables

**Table 1:** The comparison between the FE analysis and the reconstructing using inverse finite element method (iFEM). The displacements are expressed in millimeter and rotations are expressed in radian.

Loading		$u_{x}$	$v_{y}$	$w_{z}$	$\theta_{x}$	$\theta_{y}$	$\theta_{z}$
135 N	FE analysis	0.81	2.43	124.06	−0.1292	0.0039	0.0011
	IFEM	0.63	2.08	116.93	−0.0846	0.0035	0.0032
	Absolute error	0.18	0.35	7.13	0.0446	0.0004	0.0021
	Percent error	22.2%	14.4%	5.7%	34.5%	10.3%	190.9%
200 N	FE analysis	1.52	4.27	168.18	−0.2154	0.0066	0.0018
	IFEM	1.17	3.72	158.09	−0.1492	0.0058	0.0047
	Absolute error	0.35	0.55	10.09	0.0662	0.0008	0.0029
	Percent error	23.0%	12.8%	6.0%	30.7%	12.1%	161.1%

**Table 2:** Comparison of deformation along the y-axis for different time by removing the load of 20 kg loaded at the wing tip, the measured strain is obtained from the fiber Bragg grating (FBG) sensor measurement, and the actual strain is calculated from the NDI measurement with iFEM.$\;C_{Y}^{U}$ and $C_{Y}^{M}$ are, respectively, the deformation computed from the unmodified and modified strain measurements with iFEM at point C.

Time/s	Measured Strain	Actual Strain	Modified Strain	$C_{Y}^{U}/\mathrm{mm}$	$C_{Y}^{NDI}/\mathrm{mm}$	$C_{Y}^{M}/\mathrm{mm}$	Percentage Error
0.04	0.001671	0.002137	0.002183	132.01	149.16	138.89	6.7%
0.14	0.001428	0.001945	0.00187	113.07	127.52	124.97	5.2%
0.24	0.001334	0.001659	0.001603	95.32	105.29	99.72	4.5%
0.34	0.001079	0.001271	0.001261	78.48	89.13	86.29	4.2%
0.44	0.001235	0.001357	0.001391	67.86	76.11	69.70	5.3%
0.54	0.000934	0.001099	0.001087	57.46	64.37	62.79	5.6%
0.64	0.00083	0.001075	0.001035	48.95	56.28	52.86	4.5%
0.75	0.000644	0.000723	0.000714	46.53	53.30	49.95	5.1%
0.85	0.000654	0.000779	0.000742	38.67	44.32	40.75	3.9%
0.95	0.000627	0.000696	0.0007	33.83	37.78	36.58	5.2%

**Table 3:** Comparison of deformation along the *x*-axis and *z*-axis for different time by removing the load of 20 kg loaded at the wing tip.

Time/s	$C_{X}^{U}/\mathrm{mm}$	$C_{X}^{NDI}/\mathrm{mm}$	$C_{X}^{M}/\mathrm{mm}$	Percentage Error	$C_{Z}^{U}/\mathrm{mm}$	$C_{Z}^{NDI}/\mathrm{mm}$	$C_{Z}^{M}/\mathrm{mm}$	Percentage Error
0.04	1.82	1.26	1.42	12.70%	4.75	3.87	4.11	6.20%
0.14	1.54	0.89	1.03	15.73%	2.37	1.92	2.06	7.29%
0.24	1.46	0.92	0.99	7.61%	3.29	2.87	3.15	9.76%
0.34	1.27	0.75	0.82	9.33%	2.46	2.14	2.33	8.88%
0.44	1.14	0.67	0.73	8.96%	2.34	2.03	2.18	7.39%
0.54	1.08	0.56	0.65	16.07%	2.26	1.98	2.09	5.56%
0.64	1.03	0.51	0.6	17.65%	2.12	1.85	1.97	6.49%
0.75	0.89	0.47	0.52	10.64%	2.06	1.74	1.88	8.05%
0.85	0.82	0.43	0.51	18.60%	1.84	1.58	1.69	6.96%
0.95	0.78	0.37	0.44	18.92%	1.79	1.62	1.72	6.17%

**Table 4:** The maximum percentage error among the individual nodal displacements,$\;disp_{Y}^{U}$ and $\;disp_{Y}^{M}$ are, respectively, the displacement computed form the unmodified and modified strain measurements with iFEM.

Node	$disp_{Y}^{U}/\mathrm{mm}$	$disp_{Y}^{NDI}/\mathrm{mm}$	$disp_{Y}^{M}/\mathrm{mm}$	Maximum Percentage Error
1	132.01	149.06	138.97	6.7%
2	79.57	94.38	88.63	6.0%
3	41.6	55.42	52.83	4.6%
4	19.41	29.31	27.79	5.1%
5	9.78	11.5	10.96	4.7%
6	3.66	4.77	4.57	4.1%

**Table 5:** Comparison of deformation along the *y*-axis for different time, $RMS_{U}$ and $RMS_{M}$ are, respectively, the accuracy of the deformation computed form the unmodified and modified strain measurements with iFEM.

Time/s	$RMS_{U}/\mathrm{mm}$	$RMS_{M}/\mathrm{mm}$	Percentage Reduced
0.04	12.01	3.97	66.8%
0.14	11.13	3.18	71.4%
0.24	10.22	3.38	66.9%
0.34	9.42	3.71	60.5%
0.44	9.51	3.20	66.3%
0.54	9.31	3.29	64.6%
0.64	7.05	2.16	69.4%
0.75	6.21	2.03	67.2%
0.85	6.15	2.50	59.2%
0.95	5.83	2.18	62.5%

**Figure A1:**
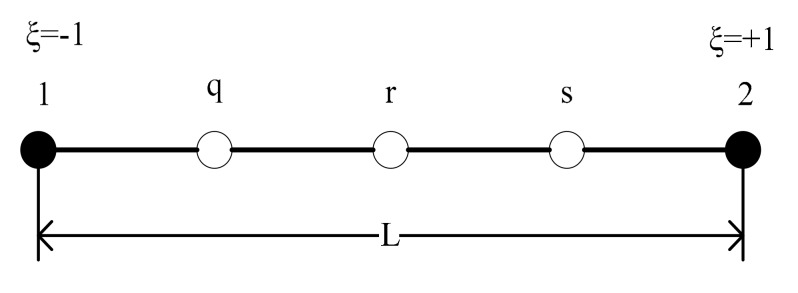
Inverse finite element geometry and nodal topology.

## Appendix A

The interpolation shape function in [4] is only applicable to constant-section beam elements. This paper also uses interdependent interpolations (Tessler and Dong, 1981) to derive the interpolation shape function suitable for the variable section beam element.

(A1) $$\begin{array}{l} u(\xi )=\sum_{i=1,r,2}L_{i}^{\left(2\right)}(\xi )u_{i}\\ v(\xi )=\sum_{j=1,q,s,2}L_{j}^{\left(3\right)}(\xi )v_{i}-\sum_{j=1,q,s,2}N_{j}^{\left(4\right)}\theta_{zj}\\ w(\xi )=\sum_{j=1,q,s,2}L_{j}^{\left(3\right)}(\xi )w_{i}+\sum_{j=1,q,s,2}N_{j}^{\left(4\right)}\theta_{yj}\\ \theta_{x}(\xi )=\sum_{i=1,r2}L_{i}^{\left(2\right)}(\xi )\theta_{xi}\\ \theta_{y}(\xi )=\sum_{j=1,q,s,2}L_{j}^{\left(3\right)}\theta_{yj}\\ \theta_{z}(\xi )=\sum_{j=1,q,s,2}L_{j}^{\left(3\right)}\theta_{zj} \end{array}$$

The shape function is expressed as follows

(A2) $$\begin{array}{l} \left[L_{1}^{\left(2\right)},L_{r}^{\left(2\right)},L_{2}^{\left(2\right)}]=\frac{1}{2}[\xi (\xi -1),2(1-\xi^{2}),\xi (\xi +1)\right]\\ \left[L_{1}^{\left(3\right)},L_{2}^{\left(3\right)}]=\frac{1}{16}((9\xi^{2}-1))[(1-\xi ),(1+\xi )\right]\\ \left[L_{q}^{\left(3\right)},L_{s}^{\left(3\right)}]=\frac{9}{16}(\xi^{2}-1)[(3\xi -1),-(3\xi +1)\right]\\ \left[N_{1}^{\left(4\right)},N_{2}^{\left(4\right)}]=\frac{L^{e}}{128}[(9\xi^{2}-1)(\xi^{2}-1),-(9\xi^{2}-1)(\xi^{2}-1)\right]\\ \left[N_{q}^{\left(4\right)},N_{s}^{\left(4\right)}]=\frac{3L^{e}}{128}[-(9\xi^{2}-1)(\xi^{2}-1),(9\xi^{2}-1)(\xi^{2}-1)\right] \end{array}$$

An element is referred to a local axial coordinate $x\in \left[0,L\right]$*,* where *L* denotes the element length. Furthermore, a non-dimensional coordinate $\xi \equiv \left(\frac{2x}{L}-1\right)\in \left[-1,1\right]$ is used to define the element shape function (Figure A1).
